# High Accuracy of Common HIV-Related Oral Disease Diagnoses by Non-Oral Health Specialists in the AIDS Clinical Trial Group

**DOI:** 10.1371/journal.pone.0131001

**Published:** 2015-07-06

**Authors:** Caroline H. Shiboski, Huichao Chen, Rode Secours, Anthony Lee, Jennifer Webster-Cyriaque, Mahmoud Ghannoum, Scott Evans, Daphné Bernard, David Reznik, Dirk P. Dittmer, Lara Hosey, Patrice Sévère, Judith A. Aberg, Oral HIV/AIDS Research Alliance, Subcommittee of the AIDS Clinical Trial Group

**Affiliations:** 1 Department of Orofacial Sciences, School of Dentistry, University of California San Francisco, San Francisco, California, United States of America; 2 Center for Biostatistics in AIDS Research, Harvard School of Public Health, Cambridge, Massachusetts, United States of America; 3 Centres GHESKIO, Port-au-Prince, Haiti; 4 Department of Dental Ecology, School of Dentistry, University of North Carolina at Chapel Hill, Chapel Hill, North Carolina, United States of America; 5 Department of Microbiology and Immunology, and Lineberger Comprehensive Cancer Center, School of Medicine, University of North Carolina at Chapel Hill, Chapel Hill, North Carolina, United States of America; 6 Center for Medical Mycology, Department of Dermatology, Case Medical Center and Case Western Reserve University, Cleveland, Ohio, United States of America; 7 Grady Health System, Atlanta Georgia, United States of America; 8 Emory University School of Medicine, Atlanta, Georgia, United States of America; 9 Social and Scientific Systems, Inc., Silver Spring, Maryland, United States of America; 10 Department of Medicine, Division of Infectious Diseases, Icahn School of Medicine at Mount Sinai, New York, New York, United States of America; 11 Department of Medicine, New York University School of Medicine, New York, New York, United States of America; King's College London Dental Institute, UNITED KINGDOM

## Abstract

**Objective:**

Many studies include oral HIV-related endpoints that may be diagnosed by non-oral-health specialists (non-OHS) like nurses or physicians. Our objective was to assess the accuracy of clinical diagnoses of HIV-related oral lesions made by non-OHS compared to diagnoses made by OHS.

**Methods:**

A5254, a cross-sectional study conducted by the Oral HIV/AIDS Research Alliance within the AIDS Clinical Trial Group, enrolled HIV-1-infected adults participants from six clinical trial units (CTU) in the US (San Francisco, New York, Chapel Hill, Cleveland, Atlanta) and Haiti. CTU examiners (non-OHS) received standardized training on how to perform an oral examination and make clinical diagnoses of specific oral disease endpoints. Diagnoses by calibrated non-OHS were compared to those made by calibrated OHS, and sensitivity and specificity computed.

**Results:**

Among 324 participants, the majority were black (73%), men (66%), and the median CD4+ cell count 138 cells/mm^3^. The overall frequency of oral mucosal disease diagnosed by OHS was 43% in US sites, and 90% in Haiti. Oral candidiasis (OC) was detected in 153 (47%) by OHS, with erythematous candidiasis (EC) the most common type (39%) followed by pseudomembranous candidiasis (PC; 26%). The highest prevalence of OC (79%) was among participants in Haiti, and among those with CD4+ cell count ≤ 200 cells/mm^3^ and HIV-1 RNA > 1000 copies/mL (71%). The sensitivity and specificity of OC diagnoses by non-OHS were 90% and 92% (for EC: 81% and 94%; PC: 82% and 95%). Sensitivity and specificity were also high for KS (87% and 94%, respectively), but sensitivity was < 60% for HL and oral warts in all sites combined. The *Candida* culture confirmation of OC clinical diagnoses (as defined by ≥ 1 colony forming unit per mL of oral/throat rinse) was ≥ 93% for both PC and EC.

**Conclusion:**

Trained non-OHS showed high accuracy of clinical diagnoses of OC in comparison with OHS, suggesting their usefulness in studies in resource-poor settings, but detection of less common lesions may require OHS.

## Introduction

Since the beginning of the AIDS epidemic, much has been learned about human immunodeficiency virus (HIV)-related oral mucosal diseases. The occurrence of oral candidiasis (OC) and hairy leukoplakia (HL), the most common HIV-related oral diseases, are strongly associated with a low CD4+ cell count [1–7] and a higher plasma viral load.[6,8] Even though the prevalence of OC, HL, and Kaposi’s sarcoma (KS) has been found to be lower among patients on antiretroviral therapy (ART),[9–18] oral warts [19–21] and salivary gland disease [19] have not decreased.

The Oral HIV/AIDS Research Alliance (OHARA) was created in 2006 to partner with the AIDS Clinical Trials Group (ACTG) Network, which plays a major role in defining the standards of care for treatment of HIV infection and related opportunistic infections.[22] OHARA’s main objectives are to investigate the oral complications associated with HIV/AIDS and potent ART as the epidemic evolves, and test novel therapies for HIV/AIDS-related oral diseases.[23] The OHARA infrastructure comprises: the Epidemiologic/Clinical Sciences Research Unit at the University of California San Francisco (UCSF), the Medical Mycology Unit at Case Western Reserve University, and the Virology Research Unit at the University of North Carolina Chapel Hill (UNC-CH).

Several ACTG protocols incorporate OHARA substudies and have shared oral end-points known to be associated with HIV/AIDS, that are assessed by non-oral-health clinical trial unit (CTU) examiners (nurses, nurse practitioners, or physicians). Even though the diagnosis of pseudomembranous candidiasis (PC), routinely referred to as “thrush”, is very familiar to HIV clinicians, the diagnosis of erythematous candidiasis (EC) is more subtle, and may be missed in the absence of specialized training. Therefore, to standardize the measurement of oral mucosal outcomes associated with HIV/AIDS within and outside the ACTG infrastructure the OHARA clinical team developed an extensive training module. The objectives of the present study was to: 1) assess the accuracy of clinical diagnoses of HIV-related oral mucosal disease made by CTU examiners who are non-oral health specialists (OHS) compared to diagnoses made by OHS; and 2) estimate the frequency of oral mucosal disease among participants recruited as part of the ACTG within domestic and international CTUs.

## Methods

### Study Design and Population

A5254 was a cross-sectional study that enrolled HIV-1-infected adults 18 years or older with or without prior ART from five ACTG CTUs in the US and one international CTU in Haiti. Institutional Review Boards or Ethics Committees of each participating institution approved the study, and each patient gave written informed consent. Participating Institutions were: Case Western, Cleveland; Emory University, Atlanta, New York University, New York; UCSF, San Francisco; and UNC-CH, Chapel Hill; in the US, and Centres GHESKIO, Port-au-Prince, in Haiti.

At study entry, participants were stratified into four strata listed below according to their documented or screening CD4+ cell count and plasma HIV-1 viral load obtained within 60 days prior to study entry:

Stratum A: CD4+ cell count ≤ 200 cells/mm³ and plasma HIV-1 viral load > 1,000 copies/mL (target N = 200).

Stratum B: CD4+ cell count ≤ 200 cells/mm³ and plasma HIV-1 viral load ≤ 1,000 copies/mL (target N = 100).

Stratum C: CD4+ cell count > 200 cells/mm³ and plasma HIV-1 viral load > 1,000 copies/mL (target N = 30).

Stratum D: CD4+ cell count > 200 cells/mm³ and plasma HIV-1 viral load ≤ 1,000 copies/mL (target N = 30).

The intent was to optimize the enrollment of individuals with more severe immunosuppression, thus a higher likelihood of HIV-related oral lesions, to provide adequate precision to our estimates of sensitivity and specificity.

### Variables and Measures

#### CTU Examiner Training and Calibration

CTU examiners (non-OHS) received a standardized training on the performance of oral mucosal examination, and on the clinical diagnoses of specific oral disease endpoints. The training consisted of a 3-hour session that included a video of a standardized oral mucosal examination; a didactic lecture using clinical slides of oral lesions, and published case definitions for each endpoint;[24] and a hands-on session where they performed oral mucosal examinations on each other. Both pre- and post-tests were administered, which consisted of 40 images (20 per test) of oral lesions with a brief history of chief complaint. The non-OHS examiners had to make a clinical diagnosis for each case, and score 80% correct answers on the post-test to be considered calibrated. Training and post-test were repeated once/year for the duration of the study and materials were available online throughout the study.

#### OHS Calibration

The OHS included four oral medicine specialists, two general dentists, one otolaryngologist, and one hygienist. All had extensive experience managing the oral health of HIV-infected patients and in diagnosing HIV-related oral diseases. The OHS watched the same presentation as that administered to the CTU examiners. Although a pre-test was not administered (since they were trained specialists) oral health specialists were asked to complete a similar post test comprised of 50 oral lesions slides as described above. Again, a minimum score of 80% was required for calibration.

#### Data Collection

Information regarding socio-demographic characteristics and general health-related variables, such as history of AIDS-related illnesses and current medications, were collected using a questionnaire administered during the study visit.

An extra-oral examination of the major salivary glands, and oral mucosal examination were performed by both a CTU examiner (non-OHS) and an OHS on each participant. Both examiners recorded their findings including descriptors of lesions with respect to location, color, and character, and a presumptive diagnosis. Examiners were blinded to each other’s findings. Oral disease endpoints explored included PC; EC; AC; HL; herpes labialis; recurrent intra-oral herpes simplex; warts; recurrent aphthous stomatitis; necrotizing gingivitis/periodontitis; necrotizing stomatitis; KS; non-Hodgkin’s lymphoma; squamous cell carcinoma; and salivary gland disease (as defined by presence/absence of parotid enlargement). A 5-minute unstimulated whole saliva (UWS) flow rate was recorded, and collected. A 1-minute oral rinse/throat wash using 10 mL of sterile saline was also collected. Both saliva and throat wash specimens were processed, frozen in aliquots at minus 80°C at the site laboratory, and shipped to the UNC-CH specimen bank unit. Before, the throat wash was processed at the sites, 2.5 mL was extracted and cultured for the presence of *Candida*. A blood draw was performed at the time of the visit for CD4+ cell count and plasma HIV-1 viral load to be measured. The CD4+ cell count and the HIV-1 viral load assay were performed in a CLIA certified laboratory for US sites, and in a laboratory certified for protocol testing by the DAIDS Immunology Quality Assurance (IQA) Program for the Haiti site. Plasma HIV-1 viral load were performed utilizing the Abbott Realtime HIV-1 Assay.

#### *Candida* cultures

The 2.5 mL of oral/throat wash was centrifuged for 5 min at 3000 rpm and the supernatant discarded. The remaining pellet was resuspended by gentle tapping, with colony counts performed from the suspension and from a 1:10 diluted suspension using a calibrated 10μL loop. Cultures were performed on Sabouraud or Potato Dextrose agar plates and incubated at 37° for 48 hrs. A culture was defined as positive, and confirming the clinical diagnosis of OC, among individuals with clinical features of OC and a number of colony forming units (CFU) ≥ 1/mL.

Resultant yeast colonies were subcultured onto an agar slant and sent to the Center for Medical Mycology, Cleveland, OH for identification. Strains were subsequently identified to genus and species using the BioMerieux API 20C system (these results are reported in a separate manuscript focusing on the microbiology aspect of the study).

### Statistical Analyses

Sample characteristics were summarized using proportions for categorical variables, and median with 1^st^ and 3^rd^ quartiles (Q1 and Q3) for continuous variables, and are presented for all CTUs, and for US and non-US sites separately. We compared CD4+ cell count and plasma HIV-1 viral load among participants in US versus non-US sites using the Wilcoxon rank-sum test.

We computed the frequency of specific oral lesions, parotid enlargement, and salivary hypofunction (defined as UWS flow rate < 0.1 mL/min) [25] by CD4+ cell count/plasma HIV-1 viral load stratum (strata C and D were combined), and explored the difference across strata using the Fisher’s Exact test. The oral lesion and parotid enlargement frequencies reported are those estimated by the OHS. The accuracy for each oral disease diagnosis by CTU examiners (non-OHS) was evaluated using sensitivity and specificity. Sensitivity was defined as the proportion of participants with an oral lesion diagnosed by CTU examiners among those with the oral lesion diagnosed by OHS. Specificity was defined as the proportion of participants without an oral disease diagnosed by CTU examiners among those without the oral lesion diagnosed by OHS.

We computed the percentage of clinical diagnosis for each type of OC that were confirmed by a positive culture (≥ 1 CFU/mL) of an oral/throat rinse aliquot.

## Results

### OHS and CTU Examiner (non-OHS) Calibration

Seven OHS participated in the calibration across the 5 US CTUs. Prior to study initiation the median score obtained from the post-test for 6 OHS was 96% (range 92–98%), compared to one OHS who served as the reference standard. After one year, the OHS from Haiti was added to the group (the Haiti CTU was delayed in its participation in the protocol due to longer IRB formalities). The calibration at 1-year after study start-up was performed for 7 OHS (6 as repeat and 1 de-novo calibration) compared to the reference standard, and the median score obtained from the post-test was 98% (range: 90–100%).

A total of 18 CTU examiners (non-OHS) participated in the baseline training, and the median post-test score obtained was 90% (range 75–100%). One examiner had a score of 75%, and after re-taking the training and post-test, scored 95%. Among the 18 examiners who were trained and calibrated, only 9 actually participated in the protocol all of whom repeated the training and calibration after one year, achieving a median score of 98% (range 85–100%).

### Sample Characteristics

Protocol A5254 enrolled its first participant in October 2009, and last participant in September 2012. After an interim power calculation revealed that the OC prevalence was higher than expected, the Study Monitoring Committee recommended closing the study to accrual before the target sample size of 360 was reached. The final sample size was 328 participants. Among the 328, one participant left the clinic before the oral examination could be performed, and 3 participants were seen by the CTU examiner, but not by the OHS, resulting in a final sample of 324 participants who received oral examinations by both examiners. Among these, the majority were Black (73%) including 58% of participants in US sites, and had never used injection drugs (85%; Table 1). The women-to-men ratio was approximately 1/5 in US sites, but 3/2 in the Haiti site. The median age was 44 years (range 19–77 years), and was similar in US and non-US sites. Among the 211 participants from US sites, 63 (30%) were enrolled to stratum A (CD4+ cell count ≤ 200 cells/mm^3^ and plasma HIV-1 viral load > 1,000 copies/mL), while 105 (93%) among Haitian participants were enrolled to stratum A. So the median CD4+ cell count was substantially lower in Haiti (71 cell/mm^3^ [Q1: 31; Q3: 136]) than in US sites (165 [Q1: 108; Q3: 296]). Similarly, the plasma HIV-1 viral load was much higher among Haitian than US participants. A Wilcoxon test comparing CD4+ cell count and plasma HIV-1 viral load between US and non-US sites yielded a p-value < 0.001 for both variables. Sixty six percent of all participants were on ART at study entry, and 8% had a history of an AIDS-defining illness. The proportion of Haitian participants receiving ART (53%) was slightly lower than US participants (73%), but the reported history of an AIDS-defining illness was similar in Haiti and US sites (5% and 9%, respectively).

**Table 1:** Socio-demographic characteristics and CD4+ cell count at enrollment, by recruitment site among 324 participants in ACTG/OHARA^1^ protocol 5254.

Sample Characteristics	All Sites	US Sites^2^	Non-US Site^2^
	(N = 324)	(N = 211)	(N = 113)
**Race; n (%)**			
White non-Latino	52 (16)	52 (25)	0 (0)
Black non-Latino	236 (73)	123 (58)	113 (100)
Latino (regardless of race)	29 (9)	29 (14)	0 (0)
Other	7 (2)	7 (3)	0 (0)
**Sex; n (%)**			
Women	111 (34)	46 (22)	65 (56)
Men	213 (66)	165 (78)	48 (42)
**Injection drug use; n (%)**			
Never used	274 (85)	161 (76)	113 (100)
Current use	4 (1)	4 (2)	0 (0)
Past use	46 (14)	46 (22)	0 (0)
**Age (years); Median [range]**	44 [19; 77]	46 [19; 73]	41 [23; 77]
**CD4+ cell count (cells/mm^3^); Median [1^st^; 3rd quartile]**	138 [75; 185]	165 [108; 296]	71 [31; 136]
**Plasma HIV-RNA load (Log_10_ copies/mL); Median [1^st^; 3^rd^ quartile]**	4.30 [1.70; 5.15]	2.06 [1.68; 4.59]	5.26 [4.81; 5.59]
**Currently on antiretroviral therapy; n (%)**	214 (66)	154 (73)	60 (53)
**History of an AIDS defining illness**	26 (8)	20 (9)	6 (5)

^1^ The Oral HIV/AIDS Research Alliance (OHARA) is a Collaborative Science Group within the AIDS Clinical Trial Group (ACTG)

^2^ **US sites**: Case Western Reserve University (Cleveland); Emory University (Atlanta); New York University (New York City); University of California San Francisco (San Francisco); University of North Carolina at Chapel Hill (Chapel Hill). **Non-US site**: Centre GHESKIO (Port au Prince, Haiti)

### Oral Disease Frequency

The overall frequency of oral mucosal disease diagnosed by OHS was 60% (Table 2). A clinical diagnosis of OC was made by OHS in 47% of participants, with a significantly higher prevalence in stratum A (71%) compared to stratum B (22%) and strata C and D combined (22%; *p <* .*0001*). The predominant type of OC was EC (60% among stratum A participants) followed by PC. The next most common oral lesion was HL (12%) followed by KS (10%), although KS was more frequently detected in participants in stratum A (17%) than HL (14%). Oral warts were detected in 8% of participants. While most oral mucosal lesions were significantly more common among participants in stratum A, parotid enlargement was seen in a significantly higher proportion of participants with CD4+ cell count > 200 cells/mm^3^ (14%) than among those with CD4+ cell count ≤ 200 cells/mm^3^ (stratum B: 11% and stratum A: 4%). Interestingly though, the frequency of salivary hypofunction (UWS flow rate < 0.1 mL/min) was significantly higher in stratum A (10%), than in Stratum B (1%) or C and D combined (5%). Finally, the frequency of oral mucosal disease diagnosed by OHS among participants in US sites was 43% versus 90% among participants in Haiti (Table 3). Specifically, the frequency of PC, EC, and KS was significantly higher among participants in Haiti than among those in US sites, which was not surprising given the lower median CD4+ cell count and higher plasma HIV-1 viral load among Haitian participants. Inversely, the frequency of parotid enlargement and oral warts was significantly lower among the Haitian participants than among participants who enrolled in US sites.

**Table 2:** Frequency of HIV-related oral lesions diagnosed by oral health specialists by CD4+ cell count and plasma HIV RNA viral load among 324 participants in ACTG/OHARA^1^ protocol 5254.

	CD4≤ 200^2^ and VL > 1000^3^	CD4 ≤ 200^2^ and VL ≤ 1000^3^	CD4 > 200^2^ and any VL^3^	*P*-value (Fishers Exact)	Total
Oral Lesion Type	N = 168; n (%)	N = 96; n (%)	N = 60; n (%)		N = 324
**Oral mucosal lesion, any type**	136 (81)	35 (36)	22 (37)	< 0.0001	193 (60)
**Oral candidiasis, any type**	119 (71)	21 (22)	13 (22)	< 0.0001	153 (47)
**Pseudomembranous candidiasis**	68 (40)	10 (10)	7 (12)	< 0.0001	85 (26)
**Erythematous candidiasis**	100 (60)	15 (16)	10 (17)	< 0.0001	125 (39)
**Angular cheilitis**	31 (18)	7 (7)	4 (7)	0.01	42 (13)
**Hairy leukoplakia**	24 (14)	5 (5)	10 (17)	0.03	39 (12)
**Kaposi sarcoma (presumptive diagnosis)**	29 (17)	2 (2)	0	< 0.0001	31 (10)
**Oral wart**	14 (8)	7 (7)	4 (7)	0.92	25 (8)
**Recurrent aphthous ulceration**	6 (4)	5 (5)	2 (3)	0.74	13 (4)
**Other HIV-related oral lesions** ^**4**^	7 (4)	4 (4)	2 (3)	1.0	13 (4)
**Parotid enlargement**	6 (4)	11 (11)	8 (14)	0.009	25 (8)
**Salivary hypofunction (UWS flow rate < 0.1 mL/min)** ^**5**^	16 (10)	1 (1)	3 (5)	0.01	20 (6)

^**1**^ The Oral HIV/AIDS Research Alliance (OHARA) is a Collaborative Science Group within the AIDS Clinical Trial Group (ACTG)

^**2**^ CD4 cell count in cells/mm^3^

^**3**^ VL: Plasma HIV-1 viral load in copies/mL

^**4**^ Other oral lesions include recurrent oral herpes simplex infection, ulcerations not otherwise specified, and necrotizing ulcerative periodontitis and gingivitis

^5^ UWS: Unstimulated whole saliva

**Table 3:** Frequency of HIV-related oral lesions diagnosed by oral health specialists by site (US sites versus non-US site) among 324 participants in ACTG/OHARA^1^ protocol 5254.

	US Sites^2^	Non-US site (Haiti)^2^	*P*-value (Fisher’s Exact)
Oral lesion type	N = 211; n (%)	N = 113; n (%)	
**Any Oral Lesion**	91 (43)	102 (90)	< 0.0001
**Oral candidiasis**	64 (30)	89 (79)	<0.0001
**Pseudomembranous candidiasis**	31 (15)	54 (48)	<0.0001
**Erythematous candidiasis**	42 (20)	83 (73)	<0.0001
**Angular cheilitis**	25 (12)	17 (15)	0.49
**Hairy leukoplakia**	21 (10)	18 (16)	0.15
**Kaposi sarcoma**	2 (<1)	29 (26)	<0.0001
**Oral wart**	21 (10)	4 (4)	0.05
**Parotid enlargement**	22 (10)	3 (3)	0.007

^1^ The Oral HIV/AIDS Research Alliance (OHARA) is a Collaborative Science Group within the AIDS Clinical Trial Group (ACTG)

^2^ **US sites**: Case Western Reserve University (Cleveland); Emory University (Atlanta); New York University (New York City); University of California San Francisco (San Francisco); University of North Carolina (Chapel Hill). **Non-US site**: Centre GHESKIO (Port au Prince, Haiti)

### Accuracy of Oral Disease Diagnoses by CTU Examiners

The sensitivity and specificity of the diagnosis of OC by CTU examiners (non-OHS) compared to OHS was very high, estimated at 90% and 92%, respectively, for all sites combined (Table 4). Accuracy of diagnoses by non-OHS was as high for PC (sensitivity: 82% and specificity: 95%) as for EC (sensitivity: 81% and specificity: 94%). Similarly, the accuracy for the diagnosis of KS was excellent, with a sensitivity of 87% and specificity of 94%. However, the sensitivity for the diagnoses of both HL and oral warts was lower than expected at 59% and 52%, respectively, even though specificity remained high (95% and 98%). Similarly, the sensitivity for detecting parotid enlargement was also low in all sites (33%), while the specificity was very high (97%). When exploring oral disease accuracy separately in US sites and Haiti, the latter was found to have higher sensitivity than in US sites for the diagnoses of EC (87% versus 69%) and HL (78% versus 43%) by non-OHS as compared to OHS. Conversely, the sensitivity of the diagnosis of oral warts by non-OHS compared to OHS was higher in US sites (62%) than in Haiti (0%).

**Table 4:** Sensitivity and specificity of HIV-related oral diagnoses made by ACTG Clinical Trial Unit non-dental examiners as compared to reference standard diagnoses made by oral health specialists among 324 participants in ACTG/OHARA^1^ protocol 5254.

All Sites			
**Oral Lesion Type**	N^3^	Sensitivity (95% CI)^4^	Specificity (95% CI)^4^
Oral candidiasis	153	0.90 (0.85, 0.95)	0.92 (0.88, 0.96)
Pseudomembranous candidiasis	85	0.82 (0.74, 0.90)	0.95 (0.92, 0.98)
Erythematous candidiasis	125	0.81 (0.74, 0.88)	0.94 (0.91, 0.98)
Angular cheilitis	42	0.69 (0.55, 0.83)	0.98 (0.96, 1.00)
Hairy leukoplakia	39	0.59 (0.44, 0.74)	0.95 (0.93, 0.98)
Kaposi sarcoma	31	0.87 (0.75, 0.99)	0.94 (0.91, 0.96)
Oral wart	25	0.52 (0.32, 0.72)	0.98 (0.97, 1.00)
Parotid enlargement	25	0.33 (0.16, 0.55)	0.97 (0.95, 0.99)
US Sites^2^			
**Oral Lesion Type**	N^3^	Sensitivity (95% CI)^4^	Specificity (95% CI)^4^
Oral candidiasis	64	0.84 (0.73, 0.92)	0.96 (0.91, 0.98)
Pseudomembranous candidiasis	31	0.84 (0.66, 0.95)	0.98 (0.95, 1.00)
Erythematous candidiasis	42	0.69 (0.53, 0.82)	0.99 (0.96, 1.00)
Angular cheilitis	25	0.76 (0.55, 0.91)	0.97 (0.94, 0.99)
Hairy leukoplakia	21	0.43 (0.22, 0.66)	0.98 (0.95, 1.00)
Kaposi sarcoma	2	1.00 (0.16, 1.00)	0.98 (0.95, 0.99)
Oral wart	21	0.62 (0.38, 0.82)	0.97 (0.94, 0.99)
Parotid enlargement	22	0.33 (0.15, 0.57)	0.97 (0.94, 0.99)
Non-US Site (Haiti)			
**Oral Lesion Type**	N^3^	Sensitivity (95% CI)^4^	Specificity (95% CI)^4^
Oral candidiasis	89	0.94 (0.87, 0.98)	0.71 (0.49, 0.87)
Pseudomembranous candidiasis	54	0.81 (0.69, 0.91)	0.85 (0.73, 0.93)
Erythematous candidiasis	83	0.87 (0.78, 0.93)	0.70 (0.51, 0.85)
Angular cheilitis	17	0.59 (0.33, 0.82)	0.98 (0.93, 1.00)
Hairy leukoplakia	18	0.78 (0.52, 0.94)	0.89 (0.81, 0.95)
Kaposi sarcoma	29	0.86 (0.68, 0.96)	0.82 (0.72, 0.90)
Oral wart	4	0.00 (0.00, 0.60)	1.00 (0.97, 1.00)
Parotid enlargement	3	0.33 (0.01, 0.91)	0.97 (0.92, 0.99)

^1^ The Oral HIV/AIDS Research Alliance (OHARA) is a Collaborative Science Group within the AIDS Clinical Trial Group (ACTG)

^2^ **US sites**: Case Western Reserve University (Cleveland); Emory University (Atlanta); New York University (New York City); University of California San Francisco (San Francisco); University of North Carolina (Chapel Hill). **Non-US site**: Centre GHESKIO (Port au Prince, Haiti)

^3^ Number of cases diagnosed by Oral Medicine specialist

^4^ Exact 95% confidence interval

The proportion of positive *Candida* culture was high among participants with clinical features of OC of all types, and for both diagnoses made by OHS and non-OHS (Table 5). For PC and EC, ≥ 93% of the clinical diagnoses made by either OHS or non-OHS were culture confirmed (CFU ≥ 1/mL in the presence or clinical signs of OC). For AC clinical diagnoses, 95% of those made by OHS and 88% of those made by non-OHS were culture confirmed.

**Table 5:** Proportion of participants with a positive oral rinse culture among 153 participants^1^ with a clinical diagnosis of oral candidiasis in ACTG/OHARA^2^ protocol 5254.

Oral candidiasis type	Oral Health Specialists		CTU examiners	
	N^3^	% positive *Candida* culture	N^3^	% positive *Candida* culture
**Pseudomembranous candidiasis**	84	94%	81	94%
**Erythematous candidiasis**	124	93%	111	94%
**Angular cheilitis**	40	95%	34	88%

^1^ All participants in the study had an oral rinse culture, however we only report the proportion of positive cultures, defined as ≥ 1 CFU/mL, among those who had clinical features of oral candidiasis, as potential confirmation of the clinical diagnosis

^2^ The Oral HIV/AIDS Research Alliance (OHARA) is a Collaborative Science Group within the AIDS Clinical Trial Group (ACTG)

^3^ Number of clinical diagnoses. Note: participants may have more than one type of oral candidiasis, so counts do not add up to 153

## Discussion

Our study revealed a much higher prevalence of OC than expected (47% in all strata and sites combined, and 71% among participants in stratum A) given that 66% were receiving ART. The prevalence of EC was especially high (1.5 times higher than that of PC). While the prevalence of OC was the highest among participants in Haiti (79%), it was also high among US participants (30%). More than 92% of the OC clinical diagnoses made by either OHS or non-OHS were culture-confirmed, assuming that in the presence of clinical features of OC a fungal culture exhibiting ≥ 1 CFU/mL for one or more *candida* specie would be considered confirmatory. The prevalence of most other oral lesions (except oral warts) was also higher in Haiti than in US sites, which is not surprising given that the median CD4+ cell count was significantly lower and the plasma HIV-1 viral load significantly higher among participants in Haiti. As previously shown in other studies, there was a strong association between a high prevalence of most oral lesions and low CD4+ cell count and detectable plasma HIV-1 viral load.[1–8] However, the prevalence of oral warts was similar in all strata, and that of parotid enlargement was actually significantly higher in the higher CD4+ cell count strata, which may explain the higher frequency of parotid enlargement and oral warts among participants in US sites compared to Haiti. This emphasizes the importance of performing oral examination even in those HIV-infected individuals who are considered “well controlled”.

The accuracy of clinical oral lesion diagnoses made by non-OHS as compared to OHS in US and non-US sites was high for the various forms of OC, which was further confirmed by the very high percentage of positive culture confirmation of the clinical diagnoses. This is an important finding, especially in resource-limited settings where OC may be a useful surrogate marker for HIV-disease stage. For example, in a recent study conducted by the OHARA/ACTG group, we found a strong association between OC and TB disease, independent of CD4+ cell count, suggesting that in resource-limited settings, OC may provide clinical evidence for increased risk of TB and contribute to TB case finding.[26] In this context it is necessary that OC be measured accurately by non-OHS such as nurses, as they are the health care providers most frequently involved with the care and screening of HIV-infected patients. To-date, few studies have examined the accuracy of oral diagnoses made by non-OHS as compared to OHS. One study among 320 HIV-infected women in Zimbabwe determined that the sensitivity of nurses’ diagnoses compared to an oral surgeon’s diagnosis of any type of OC was 73% (and the specificity 95%), but the sensitivity of nurses’ diagnoses of EC was low (51%).[27] Similarly, among 355 HIV-infected women in the Women’s Interagency HIV Study, when oral mucosal “abnormality” was used as outcome detected by non-OHS, sensitivity was 75% for PC, and 58% for EC when compared to OHS diagnoses.[28] Thus, the present study represents a great improvement with respect to the accuracy of the diagnosis of all types of OC compared to previous work, suggesting that the standardized module developed to train the non-OHS was effective with regards to OC detection. Similarly, we found a high accuracy for the clinical diagnosis of oral KS (sensitivity and specificity of 87% and 94%, respectively).

The sensitivity of non-OHS diagnoses was lower for less common oral lesions, such as HL (in US sites), oral warts, and parotid enlargement, even though the specificity remained high for these conditions. A possible explanation for the low sensitivity of less common oral lesion diagnoses made by non-OHS may be frequency of detection. There was a relatively low number of cases seen over an extended period of time (it took three years to complete the study due to the difficulty in recruiting participants with low CD4+ cell count at US sites). Despite yearly trainings and online access, the CTU examiners (non-OHS) may not have been able to retain their competency with respect to the clinical diagnosis of less common conditions. This underscores the importance of repeatedly seeing oral mucosal lesions in a patient’s mouth in addition to seeing them on a picture in learning how to make a differential diagnosis. For OC, the CTU examiners had much opportunity to see the lesions clinically, and thus to learn how to differentiate one type of OC from another, whereas for less common lesions the clinical exposure was insufficient to reinforce the training acquired from clinical pictures. The higher sensitivity for the diagnosis of HL in the Haiti site (78% versus 43% in the US site), is consistent with this explanation as HL was seen more frequently in Haiti (16%) than in domestic sites. This is a difficult issue to circumvent, because clinical training sessions would require a very large number of HIV-positive patients to be able to visualize uncommon lesions. However, there is little reason to try to address this issue since the use of oral lesions as surrogate markers of HIV stage pertains mainly to common lesions like OC.

Our group previously established case definitions for oral mucosal manifestations of HIV/AIDS in the post HAART era.[24] This is the first study to attempt to standardize training of oral mucosal disease diagnoses among non-OHS across multiple sites and countries, and to formally assess the accuracy of these diagnoses. This is also the first study to report the prevalence of oral lesions in a population with HIV-disease in Haiti. An extensive PubMed search, with the key words HIV, Haiti, and mouth, or oral, or OC, did not yield a single study describing the prevalence of oral lesions in HIV-infected populations in Haiti. In the present study, the prevalence of OC (79%), oral KS (26%), and HL (16%), were staggeringly high among Haitian participants given that 53% were receiving ART. Although despite half the Haitian participants receiving ART, the median CD4+ cell count was only 71 cells/mm^3^ and the median plasma HIV-1 viral load was 180,000 copies/mL, which certainly explains the high prevalence of oral opportunistic infections. This finding exemplifies the potential use of oral lesions as a marker of underlying immune suppression and ongoing HIV viral replication. While participants who were receiving ART initiated treatment at least 3 months prior to study entry (as required by the protocol), a subset of individuals may have been within their first 6 months of treatment, and may not have yet achieved optimal immune reconstitution leaving them vulnerable to opportunistic infections. The high prevalence of oral lesions among HIV-positive patients in Haiti and their persistence in the US HIV-positive populations underscores the importance of medical providers performing routine oral examinations as part of their physical examinations of HIV-infected patients. Training in the diagnosis and treatment of these conditions is critical, since most of these patients would likely not have access to care from an OHS.

The strengths of this study include the large number of participants, which allowed us to assess estimates of sensitivity and specificity of OC diagnosis by non-OHS as compared to OHS with high precision. Further, clinical diagnoses of OC were confirmed by culture, which is the gold standard for definitive OC diagnosis. We determined that non-OHS can be effectively trained in the recognition of not only PC (also known as thrush), but also EC, which is characterized by a discrete patchy erythema, and is often missed by non-OHS. The lower sensitivity of the non-OHS’ diagnoses of less common lesions like oral warts or HL suggests that any clinical trial or study that includes these endpoints should either rely on OHS for the diagnosis, or provide repeated training at 6-month intervals, with non-OHS spending some time in an oral medicine clinic setting where they would have the opportunity to observe these lesions. The next step for the present study is to assess the oral *Candida* carriage threshold at which patients develop clinical manifestations of OC.
